# Efficacy of Probiotic Supplementation Therapy for *Helicobacter pylori* Eradication: A Meta-Analysis of Randomized Controlled Trials

**DOI:** 10.1371/journal.pone.0163743

**Published:** 2016-10-10

**Authors:** Muhan LÜ, Shan Yu, Jiaqi Deng, Qiong Yan, Chun Yang, Guodong Xia, Xian Zhou

**Affiliations:** 1 Department of Gastroenterology, the First Affiliated Hospital of Southwest Medical University, Sichuan, People’s Republic of China; 2 School of Foreign Languages, Southwest Medical University, Sichuan, People’s Republic of China; Monash University, AUSTRALIA

## Abstract

**Background:**

Traditional *Helicobacter pylori* (*H*. *pylori*) eradication therapies have shown efficacies below 80% in several studies, and their use has been accompanied by antibiotic-related side effects. Some recent studies have reported that supplementing standard therapies with probiotics can improve the efficacy and tolerability of *Helicobacter pylori* eradication therapy.

**Objective:**

To assess the effects of probiotic supplementation on the eradication rates and therapy-related adverse event rates of anti-*Helicobacter pylori* regimens.

**Methods:**

We searched PubMed, Medline, the Cochrane Central Registry of Controlled Trials and the Chinese Biomedical Database for eligible randomized controlled trials published through July, 2015. Review Manager 5.3 was used for all statistical analyses.

**Results:**

Thirteen randomized controlled trials involving a total of 2306 patients were included in our analysis. Intent-to-treat (ITT) analysis performed using a fixed-effects model (test for heterogeneity I^2^ = 45%) showed that the pooled relative risk (RR) of eradication was significantly higher in the probiotic supplementation group than in the control group [RR 1.15, 95% confidence interval (CI): 1.10–1.20, *P*<0.00001]. The incidence of total antibiotic-related side effects was lower in the probiotic supplementation group than in the control group, and the pooled RR (studies n = 9) was 0.71 (95% CI: 0.54–0.94, *P* = 0.02), as determined using a random-effects model (heterogeneity test I^2^ = 59%). Certain adverse events, such as nausea and vomiting (RR = 0.58, 95% CI 0.35–0.95, *P* = 0.03), diarrhea (RR = 0.51, 95% CI: 0.31–0.84, *P* = 0.008) and constipation (RR = 0.47, 95% CI: 0.28–0.80, *P* = 0.005), were reported at lower rates in the probiotic supplementation group than in the control group. Subgroup analysis showed that eradication rates were significantly improved in both adults (RR = 1.14, 95% CI: 1.09–1.19, *P*<0.00001) and children (RR = 1.24, 95% CI: 1.05–1.47, *P* = 0.01) in the probiotic supplementation group and that no regional differences between Europe (RR = 1.17, 95% CI: 1.09–1.24, *P*<0.00001) and Asia were present (RR = 1.14, 95% CI: 1.06–1.22, *P* = 0.0002). However, the total adverse event rate was not decreased in the adult group (RR = 0.80, 95% CI: 0.61–1.04, *P* = 0.1) or the Asian group (RR = 0.68, 95% CI: 0.39–1.18, *P* = 0.17). Subgroup analyses examining therapy regimens and treatment durations showed that probiotic supplementation increased eradication rates in the triple-therapy (RR = 1.18, 95% CI: 1.12–1.25, *P*<0.00001), seven-day treatment (RR = 1.21, 95% CI: 1.12–1.31, *P*<0.00001) and fourteen-day treatment (RR = 1.13, 95% CI: 1.06–1.20, *P* = 0.0002) groups. The incidence of antibiotic-related side effects was significantly reduced in all groups, with the exception of the quadruple-therapy subgroup (RR = 1.13, 95% CI: 0.60–2.13, *P* = 0.07) and the fourteen-day therapy subgroup (RR = 0.96, 95% CI 0.61–1.51, *P* = 0.86). Supplementation with *Lactobacillus* alone (RR = 1.24, 95% CI: 1.12–1.38, *P*<0.0001) or multi-strain probiotics (RR = 1.12, 95% CI 1.07–1.18, *P*<0.00001) was effective at improving *H*. *pylori* eradication rates. However, supplementation with *Lactobacillus* alone did not significantly decrease the overall incidence of side effects (RR = 0.61, 95% CI: 0.11–3.51, *P* = 0.58). Our study also showed that probiotic supplementation before, during or after *H*. *pylori* eradication therapy improved eradication rates, regardless of supplementation duration. Furthermore, probiotic supplementation during *H*. *pylori* treatment reduced the incidence of side effects.

**Conclusion:**

Probiotic supplementation during anti-*Helicobacter pylori* treatment may be effective for improving *H*. *pylori* eradication rates, minimizing the incidence of therapy-related adverse events and alleviating most disease-related clinical symptoms. However, our results should be interpreted with caution because of the presence of heterogeneity across the trials included in this analysis.

## Introduction

*Helicobacter pylori*, a gram-negative and microaerophilic bacterium, was successfully isolated from the gastric mucosa of patients with chronic gastritis by Australian scientists Warren and Marshall in 1983 [1]. *H*. *pylori* infection is a serious and common public health problem, with a reported prevalence of approximately 50% worldwide [2]. In developing countries, the prevalence can rise to 80–90% [3]. Most *H*. *pylori* infections typically develop in children and exhibit a variety of clinical presentations, as some children are asymptomatic, while others present with serious diseases.

*H*. *pylori* is reportedly closely associated with chronic gastritis, as well as increased risks of peptic ulcer disease, gastric cancer, and mucosa-associated lymphoid tissue (MALT) lymphoma. The relationships between *H*. *pylori* infection and other diseases, such as unexplained iron-deficiency anemia, idiopathic thrombocytopenic purpura (ITP), and vitamin B12 deficiency [4,5], are still under investigation. Managing *H*. *pylori*-related complications requires *H*. *pylori* eradication. The most commonly prescribed standard triple-therapy regimen comprises a proton-pump inhibitor, clarithromycin, and amoxicillin/nitroimidazole. This combination has been used for decades and remains the recommended approach for *H*. *pylori* eradication [6,7]. However, eradication rates have continued to decline steadily over the last decade [8, 9]. Eradication failure rates currently exceed 20% in several countries [10], and eradication failure is closely associated with antibiotic resistance caused by antibiotic overuse or misuse [11, 12]. Therefore, new therapies or adjunctive treatments to standard eradication regimens are needed. As microorganisms, probiotics play an important role in stabilizing the intra-gastric micro-ecological environment [13]. Some recent studies have reported that several probiotic strains (especially *Lactobacillus*, *Bifidobacterium* and *Saccharomyces boulardii*) exert antagonistic effects against *H*. *pylori* both *in vitro* and *in vivo* [14], and some studies indicate that probiotic supplementation as an adjunct to eradication treatment may improve *Helicobacter pylori* eradication rates and reduce the incidence of adverse effects [15–18]. However, these results remain controversial [19]. Therefore, we conducted a meta-analysis to evaluate the role of probiotics in *H*. *pylori* eradication therapy.

## Methods

The protocol of this retrospective study was approved by the ethics committee of Southwest Medical University, which waived the requirement of written informed consent, as this study was a meta-analysis. Patient risk, privacy, welfare, and rights were given appropriate consideration. All data were analyzed anonymously. This study was carried out in accordance with the Declaration of Helsinki.

### Literature search strategy

We performed systematic searches in PubMed, Medline, the Cochrane Central Registry of Controlled Trials and the Chinese Biomedical Database (from inception until July 2015) to identify eligible trials. The following principal search terms and MESH headings were used: ‘probiotics’ or ‘probiotic’ or ‘*Lactobacillus*’ or ‘*Bifidobacterium*’ or ‘yeast’ or ‘yogurt’ and ‘*Helicobacter pylori*’ or ‘*H*. *pylori*’. We limited our searches to randomized controlled trials, clinical trials, and controlled clinical trials involving human participants that were published in English or Chinese. We also performed comprehensive manual searches of the reference lists of all returned articles, conference abstracts and key review articles to identify other relevant studies. We contacted the corresponding authors of some of the randomized studies to screen these trials further. Additionally, we searched clinicaltrails.gov to identify eligible trails that were registered but not yet published.

### Inclusion criteria

The following studies were included in our analysis: (1) randomized controlled trials using clear and adequate randomization methods, (2) studies published in either English or Chinese, (3) studies with the most recent publication date in cases of duplicate reports involving the same study population, (4) studies featuring participants with *Helicobacter pylori* infections confirmed by at least one generally accepted method (13C-urea breath test (UBT), rapid urea test (RUT), *H*. *pylori* culture, histopathology or stool antigen test) and (5) studies comparing at least two treatment groups consisting of (a) control patients who underwent antibiotic treatment with placebo or without extra interventions and (b) patients receiving identical antibiotic therapy plus probiotics. All studies were required to provide ethnicity and sample size information, as well as other sample information. The primary outcome was the *H*. *pylori* eradication rate. Eradication was defined as a negative test result at least four weeks after eradication therapy. The secondary outcomes were the overall and specific side effect rates.

### Exclusion criteria

The following studies were excluded from the analysis: (1) studies with incomplete or missing information; (2) studies using inadequate or unclear randomization methods; (3) studies including patients suffering from immunological diseases, chronic decompensated diseases, cardiovascular diseases, or upper respiratory tract infections, as well as patients who had taken proton pump inhibitors, H2 receptor antagonists, antibiotics or bismuth compounds during the preceding 2 weeks; and (4) case reports, comments, letters, and reviews.

### Data extraction

Data were extracted independently from all eligible studies by two reviewers using a prepared standardized data abstraction sheet. Disagreements were resolved via discussion or consultation with a third reviewer. We extracted the following information from each study: the first author’s name, year of publication, trial location, baseline features of the enrolled participants, initial/rechecking methods for identifying *H*. *pylori* infection, *H*. *pylori* eradication regimen, probiotic treatment type and course, follow-up time, and key outcomes recorded, including the eradication and adverse effect rates.

### Quality assessment

Quality assessments were performed with the Cochrane ‘Risk of bias’ assessment tool [20], based on the following parameters: random sequence generation, allocation concealment, blinding of participants and personnel, blinding of outcome assessments, incomplete outcome data and selective reporting. The final results of these assessments were presented as two graphs (risk of bias summary and risk of bias graph) and were generated using RevMan 5.3 statistical software. Two reviewers independently analyzed and graded each study as having a ‘low’, ‘high’ or ‘unclear’ risk of bias [21]. Consensus was reached via discussion.

### Statistical analysis

Review Manager 5.3 (RevMan) was used to perform all data analyses. Dichotomous outcomes were expressed as risk ratios (RRs) with 95% confidence intervals (CIs). *P* values <0.05 were considered statistically significant. Eradication rates and side effects incidences were assessed via intent-to-treat (ITT) principle analysis.

Heterogeneity between studies was quantified via chi-square tests and the inconsistency (I^2^) statistic. I^2^>50% and/or *P*<0.1 indicated that significant heterogeneity was present. I^2^ was also used to assess the level of heterogeneity (0–25%: homogeneity, 25–50%: low heterogeneity, 50–75%: moderate heterogeneity, >75%: high heterogeneity) [22]. Pooled estimates of efficacy were analyzed using a fixed-effects model (Mantel-Haenszel) if no heterogeneity was present [23]. Data analyses were performed using a random-effects model if significant heterogeneity was present (over 50%) [24]. We performed preplanned subgroup analyses based on the ages and locations of the participants, the probiotic species, and the eradication and probiotic regimens. We also performed exclusion sensitivity analysis to evaluate the stability of the primary outcome of this study. Potential publication bias was tested by visually inspecting the funnel plots.

## Results

### Study identification and selection

**Fig 1:**
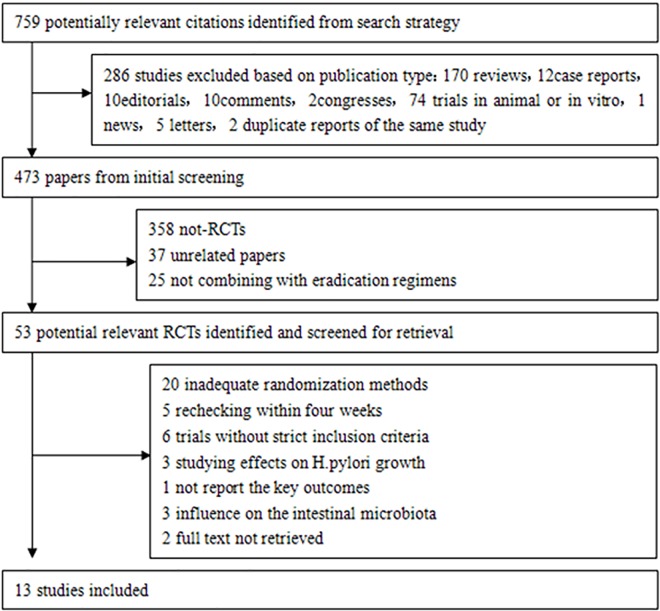
Flowchart of eligible trial selection.

Our literature search yielded a total of 759 studies, 286 of which were excluded because they were unsuitable publications. A total of 420 studies were excluded via the initial screening (358 non-RCTs, 37 unrelated papers, and 25 that did not combine probiotic supplementation with eradication therapy). We screened the remaining 53 articles for relevance and to determine if they met our inclusion criteria. Thirteen of these RCTs [25–37] met our criteria. The indicated flow diagram depicts the detailed eligible study selection process (Fig 1).

### Study characteristics

A total of 13 RCTs involving 2306 patients were included in our meta-analysis. All studies were published between 2006 and 2015 and were conducted in Asia, Europe, and South America. With the exception of one multi-center trial [25], all studies were single-center trials. The baseline characteristics of the selected studies are summarized in detail in Table 1.

**Table 1:** Baseline characteristics of the included studies.

Study(year)	Location	Patients	Total cases(Exp/Cont)	H.pylori infection diagnosis(initial/rechecking)	Eradication regimen(dose)	Regimen duration(days)	Probiotics(dose,duration,time^1^)	%Eradication rate(number of patients)(Exp/Cont)	%Side effects(number of patients)(Exp/Cont)
Hauser G^[^^25^^]^2015	Croatia(Europe)	Adults	650(333/317)	RUT,HpSA,UBT/HpSA,UBT(6weeks later)	PPI(O 20mg or P 40mg or L 30mg) bid, C 500mg bid, A 1000mg or Me 400mg bid, placebo	14	*L*.*rhamnosus*,*Bifidobacterium*,^**1**^0^8^−10^10^ per capsule bid 2weeks; a	ITT:87.38(291/333);72.55(230/317)	-/-
Ling Y^[^^26^^]^2014	China(Asia)	Adults	132(66/66)	Histology, RUT/Histology,^14^C-UBT(4weeks later)	O 20mg qd, C 500mg bid, Me 400mg tid, placebo	7	*L*.*acidophilus*2capsule,bid 1week;a	ITT:87.9(58/66);63.6(42/66)	4.5(3/66);18.2(12/66)
Wang YH^[^^27^^]^2014	China(Asia)	Children	100(49/51)	^13^C-UBT/^13^C-UBT(6weeks later)	PPI 0.6–0.8mg/kg bid, C 10–15mg/kg bid, A 30–50mg/kg bid	14	*L*.*acidophilus-5*(4.7x10^9^cfu/100g) 2g,*B*.*bifidum-12*(4.3x10^8^cfu/100g) 2g<5 years old qd 6weeks,>5 years old bid 6weeks;c	ITT:73.5(36/49);56.9(29/51)	10.2(5/49);23.5(12/51)
Francavilla R^[^^28^^]^2014	Italy(Europe)	Adults	88(44/44)	Histology, RUT/^13^C-UBT(8weeks later)	PPI standard dose bid, C 500mg bid, A 1000mg bid, Placebo	7	*L*.*acidophilus-5*(4.7x10^9^cfu/100g)2g,*B*.*bifidum-12*(4.3x10^8^cfu/100g)2g qd 1week; a	ITT:75(33/44);65.9(29/44) PP:76.7(33/43);67.4(29/43)	40.9(18/44); 61.4(27/44)
Ahmad S^[^^29^^]^2013	Iran(Asia)	Adults	180(90/90)	Histology, RUT/^13^C-UBT(4weeks later)	O 20mg bid, C 500mg bid, A 1000mg bid, Bs 240mg bid	14	*Lactobacillus strains(L*.*casei*,*L*.*rhamnosus*,*L*.*acidophilus*,*L*.*bulgaricus)and BifidobacteriumStrains(B*.*breve and B*.*longum)*10^8^cfu/per capsule bid 2weeks; a	ITT:76.7(69/90); 81.1(73/90) PP:82.1(69/84); 84.8(73/86)	18.8(17/90); 16.6(15/90)
Ahmad k^[^^30^^]^2013	Iran (Asia)	Children	66(33/33)	RUT,Histology/HpSA(4–8weeks later)	O 0.5mg/kg bid, F 3mg/kg bid, A 25mg/kg bid, placebo	7	*L*.*acidophilus*,*L*.*rhamnosusL*.*bulgaricus*,*L*.*CaseiStreptococcus thermophilus*,*B*.*infantis and B*.*Breve* 10^9^cfu/d;qd 2weeks; a	ITT:90.9(30/33); 69.7(23/33)	21.2(7/33); 63.6(21/33)
Navarro-Rodriguez^[^^31^^]^2013	Brasil(SouthAmerica)	Adults	107(55/52)	Histology, RUT,^13^C-UBT/^13^C-UBT(8 weeks later)	L 30 mg bid, Te 500 mg bid, F 200 mg bid, placebo	7	*L*.*acidophilus*(1.25x10^9^cfu),*L*.*rhamnosus*(1.25x10^9^cfu),*B*.*bifidum*(1.25x10^9^cfu),*Streptococcus faecium*(1.25x10^9^cfu)bid 30days;c	ITT:81.8(49/55);79.6(44/52) PP:89.8%(49/51);85.1(44/49)	59.3(32/55);71.2(37/52)
Manfredi M^[^^32^^] ^2012	Italy(Europe)	Adults	149(73/76)	Histology, HpSA/HpSA(8–10 weeks later)	E 20 mg, A 1000 mg bid 5days plus E 20 mg, C 500 mg, T 500 mg bid for next 5 days, placebo	10	*L*.*acidophilus*(10^9^ per bottle)*B*. *bifidum*(5x10^8perbottle)*Streptococcus thermophilus*(10^9^ per bottle) *L*.*bulgaricus*(10^9^ per bottle)bid 10 days; a	ITT:89(65/73);88.2(67/76) PP:92.9(65/70);94.4(67/71)	39.7(29/73)65.8(50/76)
Ojetti V^[^^33^^]^2012	Italy(Europe)	Adults	90(45/45)	^13^C-UBT/^13^C-UBT(6weeks later)	E 20mg bid, A 1000mg bid, Lev 500mg bid	7	*L*.*reuteri*(10^8^cfu)tid 2weeks; c	ITT:80(36/45);60(28/45)	-/-
Deguchi R^[^^34^^]^2012	Japan(Asia)	Adults	229(115/114)	RUT, Histology, culture/13C-UBT, HpSA(8weeks later)	R 10 mg bid, C 200 mg bid, A 750 mg bid	7	*L*.*gasseri* OLL2716(>10^9^ cfu/g) 112 gbid 4 weeks; b	ITT:82.6(95/115);69.3(79/114) PP:85.6(95/111);74.5(79/106)	5.2(6/115);3.5(4/114)
Yoon H^[^^35^^]^2011	Korea(Asia)	Adults	337(151/186)	RUT,Histology,^13^C-UBT/^13^C-UBT(4weeks later)	E 20mg bid, M 400mg bid, A 1000mg bid	14	*L*.*acidophilus* HY2177(>10^5^cfu/mL),*L*.*casei* HY2743(>10^5^cfu/mL),*B*.*longum* HY8001(>10^6^cfu/mL)(>10^8^cfu/mL); 150mL qd 4weeks;c	ITT:68.9(104/151);66.7(124/186) PP:86(104/121);78.5(124/158)	28.5(43/151);25.3(47/186)
Sheu BS^[^^36^^]^2006	Taiwan(Asia)	Adults	138(69/69)	Histology,^13^C-UBT/^13^C-UBT(6weeks later)	O 20mg bid, Me 500mg bid, A 1000mg bid, Bs 120mg tid	7	*L*.*acidophilus*La5,*B*.*lactis* Bb12,*L*.*bulgaricus*,*Streptococcus thermophilus*(>10^9^bacteria/mL) 200mL bid 4weeks;b	ITT:85(59/69);71.1(49/69) PP:90.8(59/65);76.6(49/64)	-/-
Lionetti E^[^^37^^]^2006	Italy(Europe)	Children	40(20/20)	RUT,Histology,^13^C-UBT/^13^C-UBT(8weeks later)	O 1mg/kg, A 50mg/kg qd 5days plus O 1mg/kg, C 15mg/kg, T 20mg/kg qd for next 5days,placebo	10	*L*.*reuteri* ATCC55730(10^8^cfu) qd 20days;c	ITT:85(17/20);80.0(16/20)	-/-

E: Esomeprazole, O: Omeprazole, L: Lansoprazole, R: Rabeprazole, T: Tinidazole, Me: Metronidazole, C: Clarithromycin, A: Amoxicillin, Lev: Levofloxacin, M: Moxifloxacin, Te: Tetracycline, Bs: Bismuth subcitrate, F: Furazolidone

1 (a represents‘ simultaneous’, i.e., administration with the eradication regimen; b represents ‘prior’, i.e., used prior to the eradication regimen/continued until the end of the eradication regimen; c represents ‘subsequent’, i.e., beginning with eradication treatment and continuing after the end of eradication treatment/used after the eradication regimen has ended)

RUT: rapid urease test, UBT: urea breath test, HpSA: H. pylori stool antigen test, Exp: experimental group, Cont: control group, cfu: colony forming units; ITT: intent-to-treat analysis, PP: per-protocol analysis.

### Risk assessment of bias in all included trials

**Fig 2:**
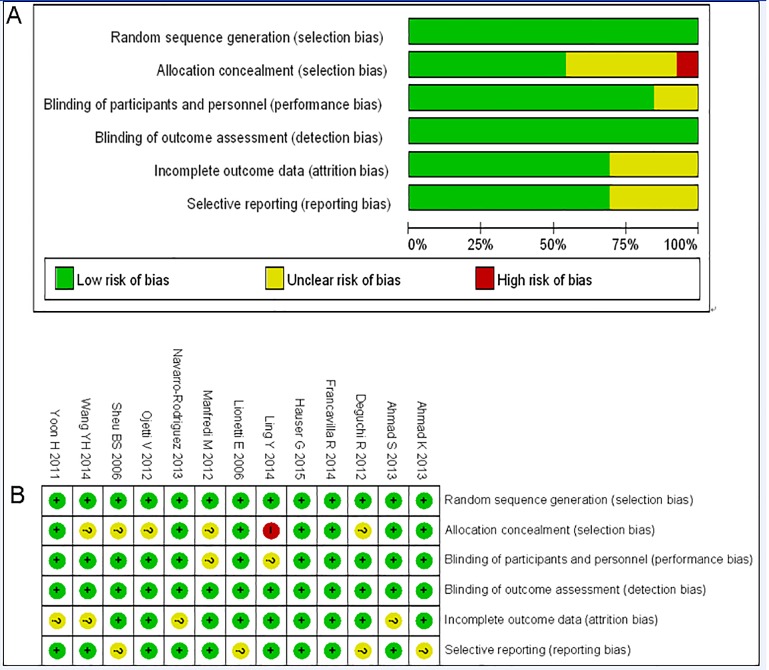
**A** risk of bias graph, **B** risk of bias summary (“+”low risk; “?”, unclear risk; “-”, high risk).

Random sequence generation (randomization method) was appropriate and clear in all included studies. Fig 2 shows the reviewers’ judgements regarding each Cochrane risk of bias item and the Cochrane risk of bias score for each citation included.

### Primary outcome: eradication rates

**Fig 3:**
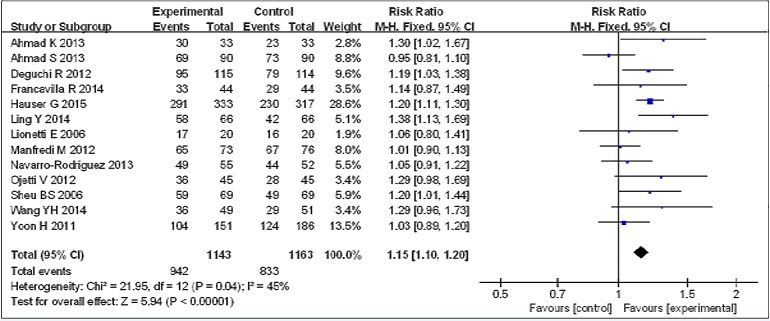
Eradication rates with or without probiotics.

Risk ratios regarding the effects of probiotic supplementation on *Helicobacter pylori* eradication rates were available for all 13 trials [25–37], which included data from 2306 patients (1,143 patients in the probiotic supplementation group and 1,163 patients in the control group). A fixed-effects model was used because significant heterogeneity was not present (I^2^ = 45%). We found that eradication rates improved by approximately 11% in the experimental group compared with the control group and that the pooled RR was 1.15 (95% CI 1.10–1.20, *P*<0.00001), as determined via ITT analysis using the fixed-effects model (Fig 3). Based on these results, we determined that probiotic supplementation can significantly increase *H*. *pylori* therapy eradication rates.

### Secondary outcomes: side effects

**Fig 4:**
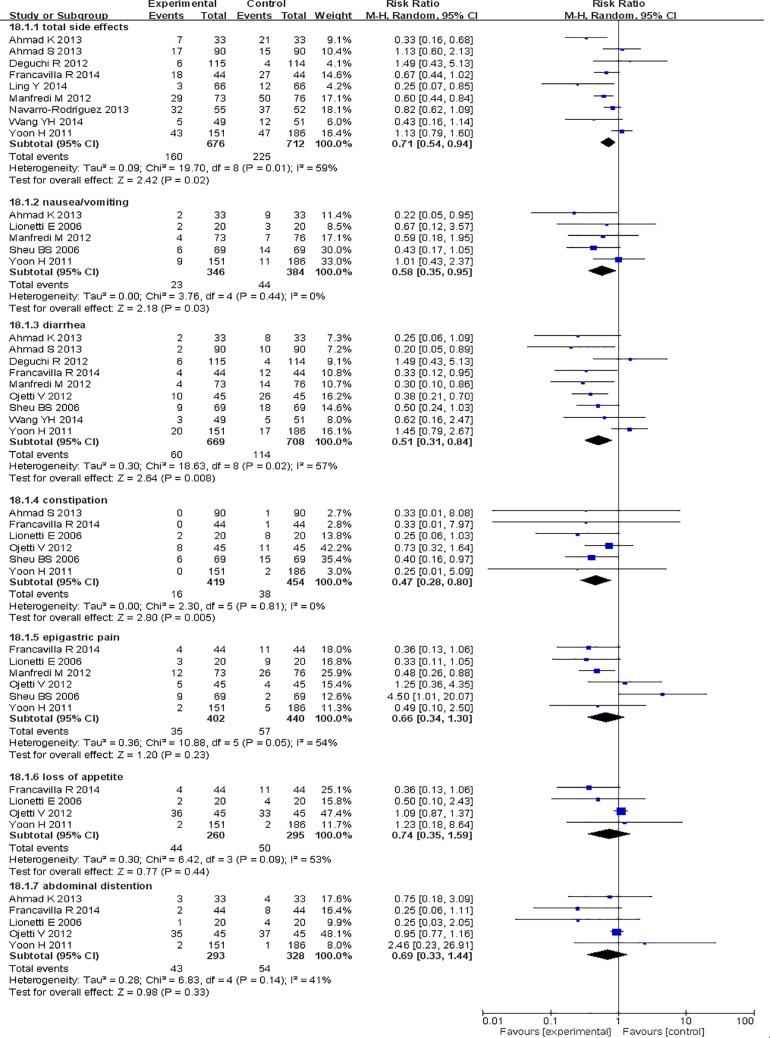
Effects of probiotic supplementation on the overall incidence of side effects and the individual incidences of specific side effects compared with the control group.

**Fig 5:**
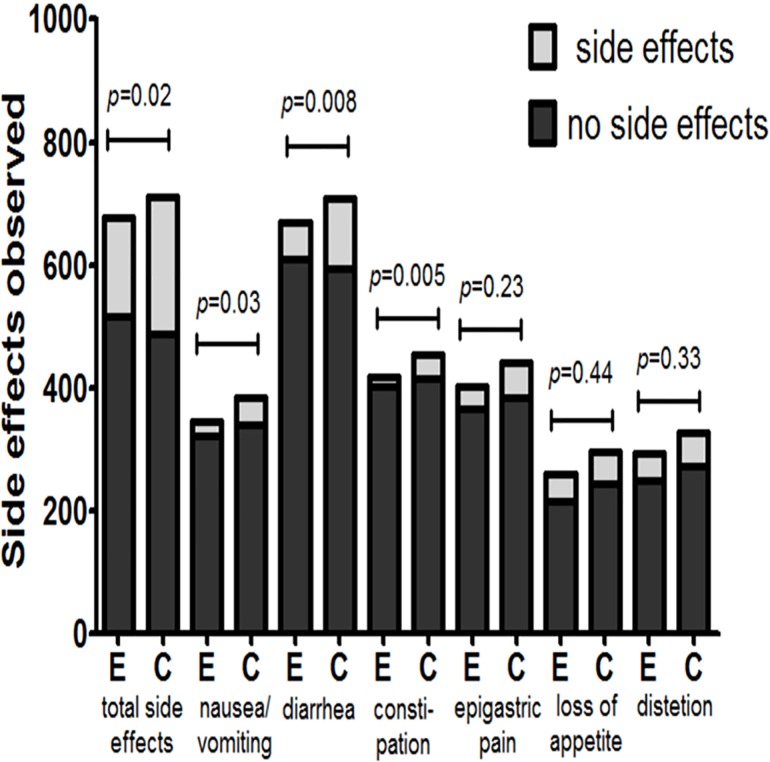
Effects of probiotic supplementation on the overall incidence of side effects and the individual incidences of specific side effects compared with the control group.

Data regarding the overall incidence of side effects were available in nine RCTs (676 participants in the supplementation group and 712 participants in the control group) and showed that the incidence of side effects was significantly lower in the probiotic supplementation group than in the control group. The overall incidence of side effects decreased by approximately 8% in the probiotic supplementation group compared with the control group. The pooled RR for the probiotic supplementation group was 0.71 (95% CI: 0.54–0.94, *P* = 0.02), as determined by ITT analysis using a random-effects model (heterogeneity test I^2^ = 59%) (Fig 4). The incidences of individual adverse events, such as nausea and vomiting, diarrhea, constipation, epigastric pain, loss of appetite, and abdominal distention, were also assessed in this meta-analysis (Figs 4 and 5). We noted significant decreases in the incidences of nausea and vomiting (RR = 0.58, 95% CI: 0.35–0.95, *P* = 0.03), diarrhea (RR = 0.51, 95% CI: 0.31–0.84, *P* = 0.008), and constipation (RR = 0.47, 95% CI: 0.28–0.80, *P* = 0.005) in the probiotic supplementation group compared with the control group. However, we noted no significant decreases in the incidences of epigastric pain (RR = 0.66, 95% CI: 0.34–1.30, *P* = 0.23), loss of appetite (RR = 0.74, 95% CI: 0.35–1.59, *P* = 0.44), or abdominal distention (RR = 0.69, 95% CI: 0.33–1.44, *P* = 0.33) in the probiotic supplementation group compared with the control group.

### Subgroup analysis

**Fig 6:**
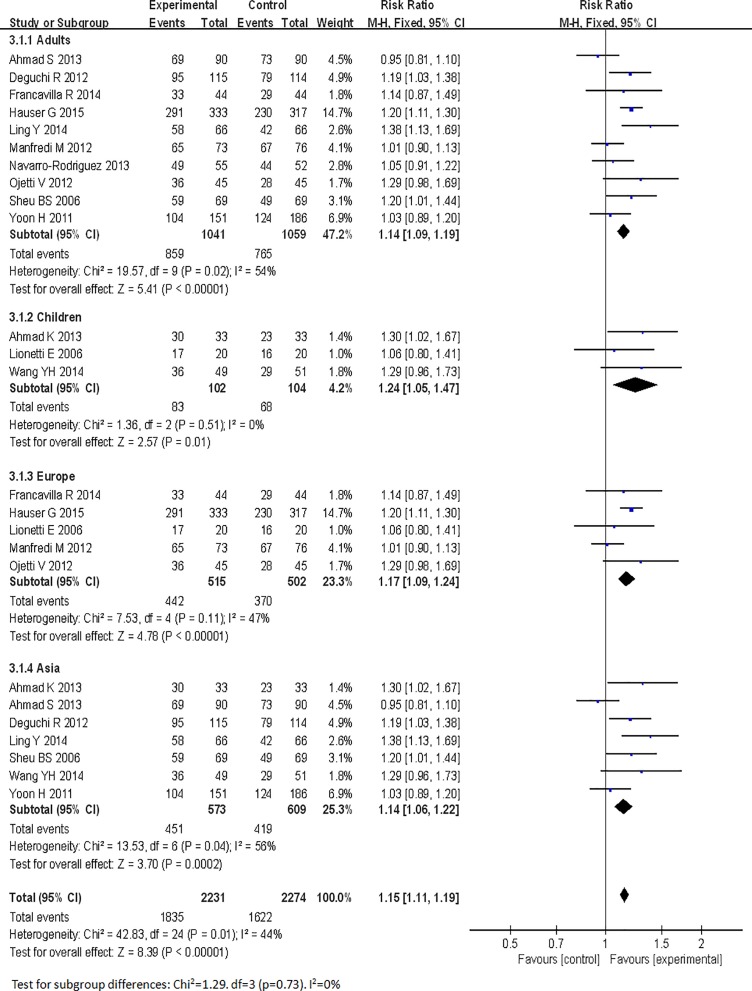
Meta-analysis of eradication rates according to age and region.

**Fig 7:**
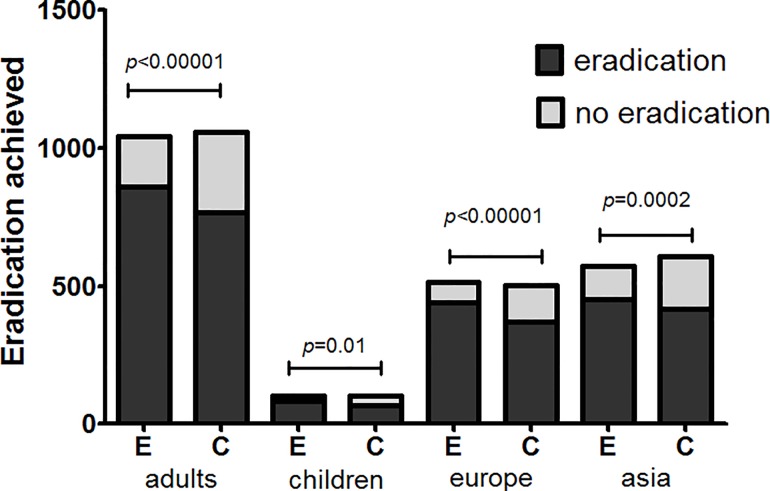
Eradication achieved according to age and region.

**Fig 8:**
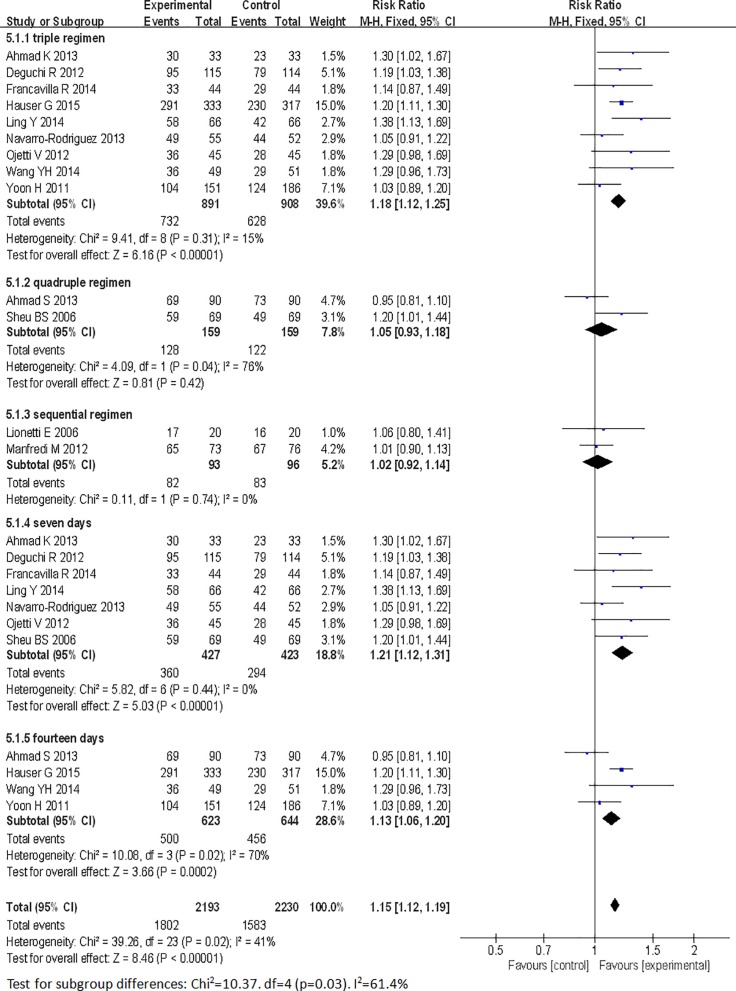
Meta-analysis of eradication rates according to eradication therapy regimen and duration.

**Fig 9:**
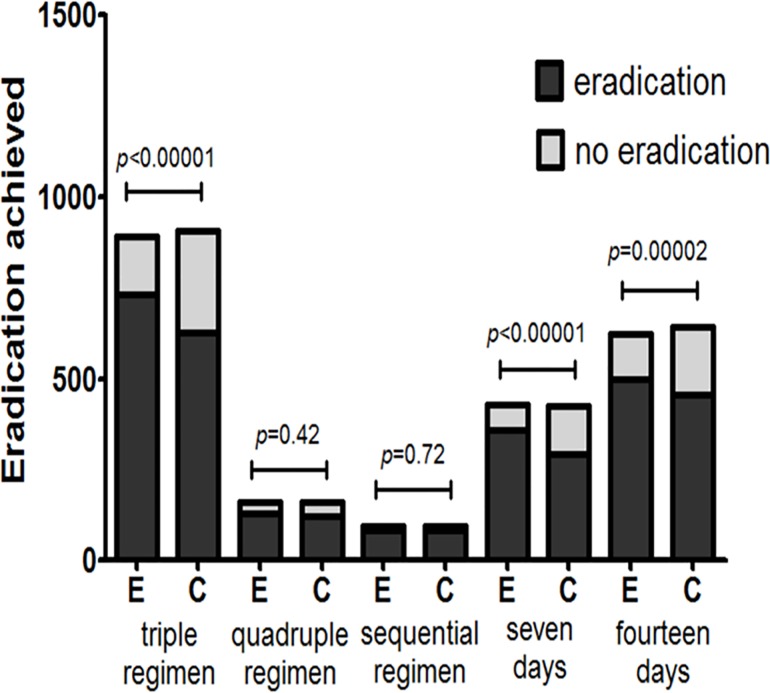
Eradication achieved by different eradication therapy regimens and durations.

**Fig 10:**
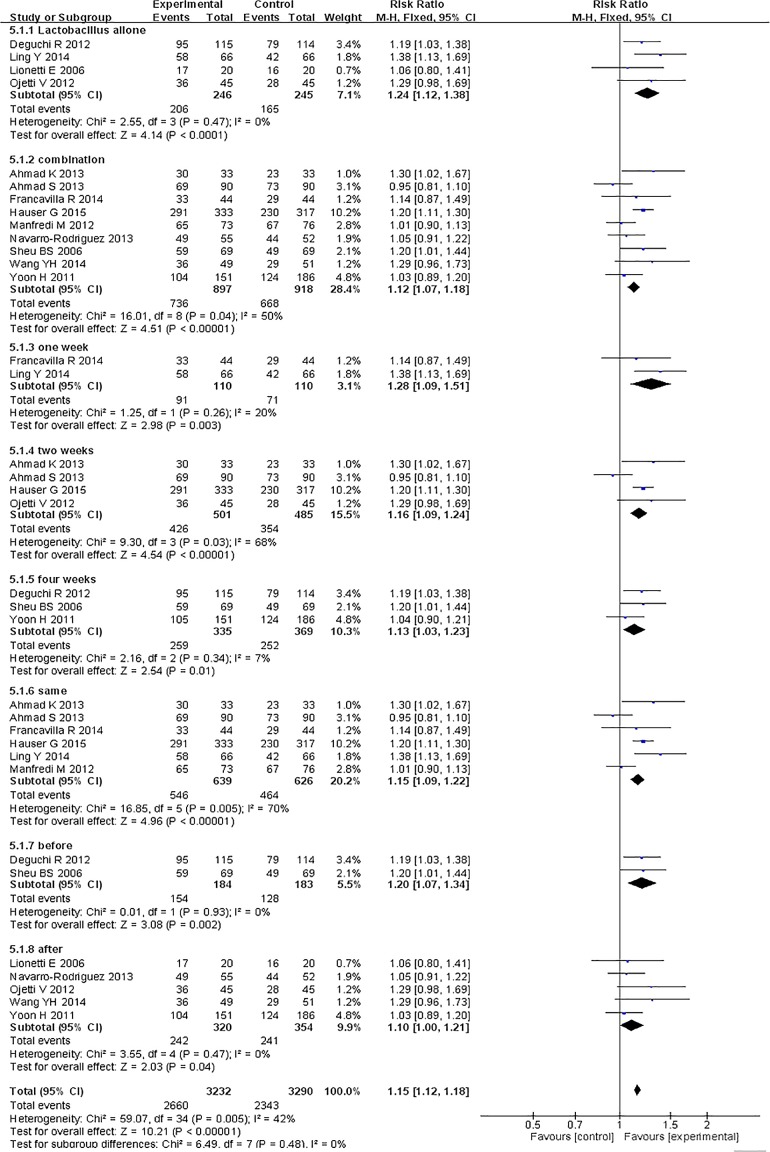
Meta-analysis of eradication rates according to probiotic species, probiotic supplementation durations and probiotic supplementation time.

**Fig 11:**
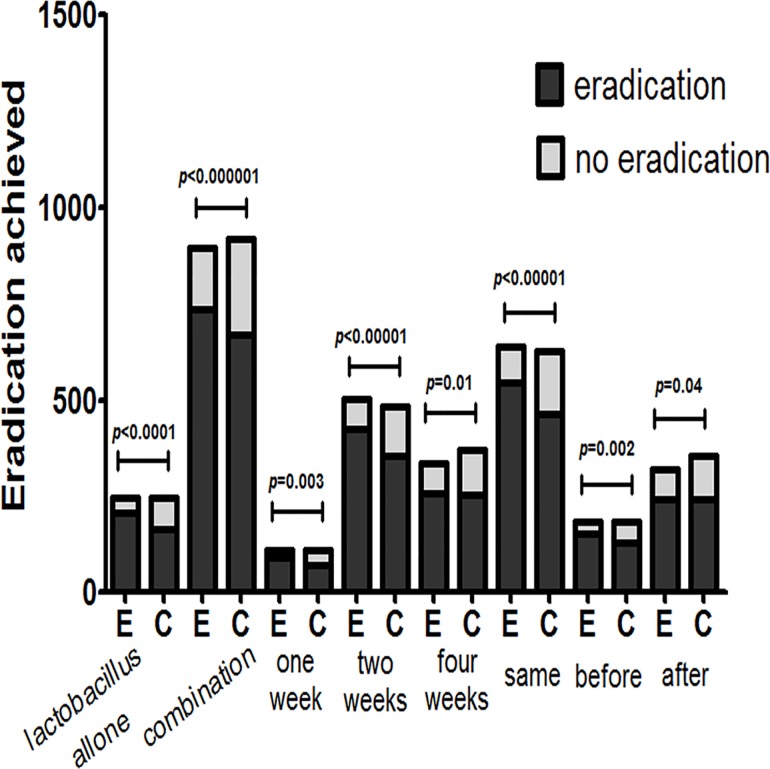
Eradication achieved by different probiotic species, probiotic supplementation durations and probiotic supplementation times.

Regarding the outcomes of eradication rates and overall side effect rates, we conducted subgroup analyses in this meta-analysis based on age (adults or children), location (Europe or Asia), eradication regimen (triple- or quadruple- or sequential therapy), eradication therapy duration (seven or fourteen days), probiotic species (*Lactobacillus* alone or multi-strain probiotics), probiotic supplementation duration (one week, two weeks, or four weeks), and probiotic incorporation time (prior to, simultaneously with or subsequent to standard treatment). The age and the location subgroup analyses showed that the adult and children subgroups exhibited significantly improved eradication rates (pooled RR = 1.14, 95% CI: 1.09–1.19, *P*<0.00001; RR = 1.24 95% CI: 1.05–1.47, *P* = 0.01, respectively) and that the pooled RRs in the Europe and Asia subgroups were 1.17 (95% CI: 1.09–1.24, *P*<0.00001) and 1.14 (95% CI 1.06–1.22, *P* = 0.0002) (Figs 6 and 7), respectively. The treatment regimen and eradication therapy duration subgroup analysis showed that probiotic supplementation increased eradication rates in the triple-therapy subgroup (RR = 1.18, 95% CI 1.12–1.25, *P*<0.00001), the seven-day eradication therapy subgroup (RR = 1.21, 95% CI: 1.12–1.31, *P*<0.00001) and the fourteen-day eradication therapy subgroup (RR = 1.13, 95% CI: 1.06–1.20, *P* = 0.0002), but not in the quadruple-therapy subgroup (RR = 1.05, 95% CI: 0.93–1.18, *P* = 0.42) or the sequential therapy subgroup (RR = 1.02, 95% CI 0.92–1.14, *P* = 0.72) (Figs 8 and 9). Subgroup analysis of specific probiotic species showed that *Lactobacillus* alone and multi-strain probiotics used as adjuncts to eradication therapy both improved eradication rates (pooled RR = 1.24, 95% CI: 1.12–1.38, *P*<0.0001; RR = 1.12, 95% CI: 1.07–1.18, *P*<0.00001, respectively) (Figs 10 and 11). Probiotic supplementation duration sub-analysis showed that the pooled RR in the one-week subgroup was 1.28 (95% CI: 1.09–1.51, *P* = 0.003), the pooled RR in the two-week subgroup was 1.16 (95% CI: 1.09–1.24, *P*<0.00001), and the pooled RR in the four-week subgroup was 1.13 (95%CI: 1.03–1.23, *P* = 0.01) (Figs 10 and 11). Subgroup analysis of the probiotic incorporation time-point showed that the pooled RR in the ‘simultaneously’ subgroup was 1.15 (95% CI: 1.09–1.22, *P*<0.00001), the pooled RR in the ‘prior’ subgroup was 1.20 (95% CI: 1.07–1.34, *P* = 0.002), and the pooled RR in the ‘subsequent’ subgroup was 1.10 (95% CI: 1.00–1.21, *P* = 0.04) (Figs 10 and 11).

Significant decreases in the incidences of therapy-related adverse events were observed in the children subgroup, the multi-strain probiotics subgroup, the triple-therapy subgroup, the sequential therapy subgroup, the Europe subgroup, the seven-day eradication therapy subgroup and the simultaneous probiotic administration subgroup (Table 2).

**Table 2:** Subgroup analysis results regarding eradication side effects.

Subgroup		Objects	Meta-analysis of overall side effects		
			relative risk(95%CI)	*P*-value	Heterogeneity (I^2^)
Age	Adults	7	0.80 (0.61,1.04)	0.1	53%
	Children	2	0.37 (0.21,0.65)	0.0005	0%
Location	Europe	2	0.63(0.48, 0.81)	0.0004	0%
	Asia	6	0.68(0.39, 1.18)	0.17	70%
Eradication regimens	Triple regimen	7	0.68 (0.47, 0.97)	0.03	63%
	Quadruple regimen	1	1.13 (0.60, 2.13)	0.7	-
	Sequential regimen	1	0.60 (0.44, 0.84)	0.002	-
Duration of eradication therapy	Seven days	5	0.61 (0.39, 0.95)	0.03	61%
	Fourteen days	3	0.96 (0.61, 1.51)	0.86	42%
Species of probiotics	Lactobacillus alone	2	0.61 (0.11, 3.51)	0.58	75%
	Combination	7	0.74(0.56, 0.97)	0.03	60%
Duration of probiotic supplementation	One week	2	0.48 (0.18, 1.25)	0.13	60%
	Two weeks	2	0.62 (0.19, 2.06)	0.44	84%
	Four weeks	2	1.15 (0.82, 1.62)	0.42	0%
Probiotic supplementation time	Simultaneous	5	0.59 (0.41, 0.86)	0.006	54%
	Prior	1	1.49 (0.43, 5.13)	0.53	-
	Subsequent	3	0.87 (0.60, 1.24)	0.44	53%

### Publication bias

**Fig 12:**
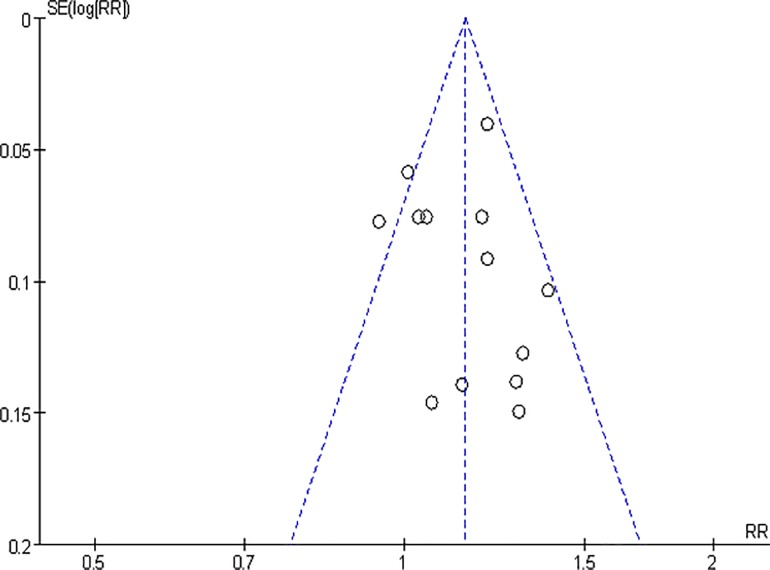
Funnel plot of the eradication rates of the included studies. E: experimental group; C: control group; Same: probiotics administered with the eradication regimen; Before: probiotic administered before the eradication regimen/continuing until the end of the eradication regimen; After: probiotic administered beginning with eradication treatment and continuing after the end of eradication treatment/used after the eradication regimen has ended.

Visual inspection of the funnel plots revealed a slightly asymmetrical distribution for the primary outcome of eradication rates (Fig 12).

## Discussion

*H*. *pylori* infection is closely associated with chronic atrophic gastritis, peptic ulcer disease, gastric cancer, and other gastrointestinal disorders. The pathogenic mechanisms underlying *H*. *pylori* infection development include *H*. *pylori* colonization, toxin-induced gastric mucosal injury, immune response-induced gastric mucosal injury and gastrin/somatostatin imbalance-induced abnormal gastric acid secretion following *H*. *pylori* infection [38]. Host factors, including inflammation, acid secretion, oxidation and *H*. *pylori* immune responses, also contribute to the pathogenesis of *H*. *pylori* infection [39]. For the past few years, the indicated eradication therapy regimen (7 days of triple-therapy consisting of clarithromycin, amoxicillin, and PPI) has been used as the first-line treatment for *H*. *pylori* infection, as recommended by the Maastricht 2–2000 Consensus Report [40]. However, unsatisfactory *H*. *pylori* eradication rates and therapy-associated adverse events are two limitations of the above regimen. Antibiotic resistance has continued to rise due to the widespread clinical application and abuse of antibiotics, such as clindamycin, metronidazole, amoxicillin, and levofloxacin, impeding efforts to eradicate *H*. *pylori*. Moreover, the administration of PPIs and diverse antibiotics has led to the disruption of the ecological equilibrium of the gastrointestinal micro-environment, resulting in a series of adverse gastrointestinal effects. Additionally, *H*. *pylori* L-form may contribute to decreases in eradication rates in unfavorable growth environments, such as environments with reduced oxygen concentrations, environments with high alkalinity, environments with high temperatures and environments exposed to amoxicillin. All these factors decrease patient compliance and tolerance, making *H*. *pylori* infection eradication much more difficult. In light of these declining eradication rates, many studies have suggested using sequential quadruple-therapy, PPI dose increases, and extended treatment courses [41]. The consensus report of the fourth nationwide *H*. *pylori* management conference and the Maastricht IV consensus recommended using quadruple-therapy consisting of bismuth, a PPI, and two types of antibiotics as a first-line therapy regimen in regions with high clarithromycin resistance [42]. Using probiotics as a supplement to standard eradication therapy was suggested as an alternative treatment method.

As they can partially stabilize or restore physiological endogenous microflora and inhibit *H*. *pylori* growth, probiotics have recently been suggested as a new anti-*H*. *pylori* therapy option. The stomach mainly contains *Lactobacillus* in its non-acidic regions and *Saccharomyces* in its acidic regions. *H*. *pylori* infection is closely associated with *Lactobacillus* quantity, and *Lactobacillus*-containing supernatants can significantly reduce *H*. *pylori* viability independent of lactic acid concentrations. Persistent *H*. *pylori* infection affects the distribution and density of flora in the gastrointestinal tract and decreases the density of *Lactobacillus* in the stomach. Many previous studies have shown that probiotic supplementation can be effective in improving the eradication rates of anti-*H*. *pylori* therapy and decreasing the incidences of antibiotic-related side effects [17,43–46]. Probiotics may act as surrogate normal micro-flora after antibiotic therapy until resident flora recover, although the mechanism underlying this phenomenon is not completely understood [47]. Several conjectural mechanisms have been suggested to explain the effects of probiotics on *H*. *pylori* growth, as follows: (1) anti-microbial substance production: *Lactobacillus* produces bacteriocins that exert direct inhibitory effects on *H*. *pylori*. *Bifidobacterium* and Enterococcus can inhibit *H*. *pylori* viability by generating heat-stable proteinaceous compounds [48]. *Lactobacillus* and *Bifidobacterium* produce lactic and acetic acid and hydrogen peroxide to inhibit *H*. *pylori*. *Lactobacillus* can secret metabolites that inhibit *H*. *pylori* urease activity [49,50]; (2) epithelial cell adhesion site competition [50]; (3) mucosal barrier stabilization [51], which likely results from increases in mucin secretion induced by mucin gene allele mutations [50, 52]; (4) immune response regulation facilitating reductions in inflammatory chemokine expression and lymphocyte infiltration[53–55], inflammation/anti-inflammation balance promotion via the MAPK and NF-kB signaling pathways and anti-inflammation promotion via suppressor of cytokine signaling (SOCS) activation in the setting of *H*. *pylori* infection[56]; and (5) probiotic-induced sIgA secretion and mucosal immune functional enhancements. Currently, the best-studied probiotics are lactic acid bacteria, particularly *Lactobacillus sp*. and *Bifidobacterium sp*.

Overall, this meta-analysis of 13 RCTs demonstrated that probiotic supplementation can improve *H*. *pylori* eradication therapy improve eradication rates, reduce the overall incidence of antibiotic-related side effects, and decrease the individual incidences of specific symptoms compared with placebo and non-interventional strategies. The outcomes of this trial were similar to those of several previous meta-analyses [57–59]. Our results indicate that eradication rates improved by approximately 11% and that overall side effect rates decreased by approximately 8% in the probiotic supplementation group compared with the control group. Subgroup analysis showed that probiotic supplementation can significantly increase eradication rates in any age group and regional population included in this study (Fig 6). However, another analysis showed that probiotic supplementation reduced the overall incidence of side effects in only the children and European groups (Table 2). Probiotics have been used safely for treating children with acute and chronic diarrhea and for preventing necrotizing enterocolitis in pre-term infants, neonatal jaundice and infantile hepatitis integrated syndrome, eczema, food allergies, respiratory tract infections, HP infection, and inflammatory bowel disease [60–62]. Childhood flora are fragile, exhibit poor stability and low immunity, and are susceptible to natural environmental perturbations and the influences of factors such as physiology, pathology, and drugs. Thus, floral disorders arise easily in children and cause related diseases. Probiotic supplementation can significantly decrease the incidence of antibiotic-related adverse events noted in Europeans and may counter the overuse of antibiotics and high rates of *H*. *pylori* antibiotic resistance noted in Asian populations. A regional bias reportedly exists with respect to the rate of antibiotic resistance. A recent literature review regarding the prevalence of antibiotic resistance in patients infected with *Helicobacter pylori* demonstrated that Asia has a higher frequency of resistance to all antibiotics commonly used to treat *H*. *pylori* infection than Europe [63]. Our examination of *H*. *pylori* resistance to clarithromycin showed that European studies reported a 24.8% reduction in clarithromycin resistance in 2014, while Asian studies reported a 32.46% increase in clarithromycin resistance [63]. Some previous studies observed that the metronidazole resistance rate ranged from 10%-50% in developed countries but ranged from 60%-90% in developing countries [64]. *H*. *pylori* antibiotic tolerance has gradually increased [65], especially in developing countries; however, *H*. *pylori* antibiotic tolerance has increased more slowly in developed countries than in developing countries and, in some cases, has increased [66]. Antibiotic resistance is widely recognized as the most important cause of *H*. *pylori* eradication failure [67,68] and inevitably increases the incidence of eradication therapy-related side effects. Reductions in antibiotic abuse in developed countries have facilitated decreases in the incidences of adverse reactions associated with *H*. *pylori* treatment, indicating that the incidence of antibiotic-related side effects is obviously reduced via probiotic supplementation. However, antibiotic overuse and abuse in Asia have resulted in a higher incidence of adverse reactions, thereby blunting any positive effects exerted by probiotic supplementation.

Our subgroup analysis examining different *H*. *pylori* therapeutic regimens and durations showed that probiotic supplementation did not improve eradication rates in patients treated with sequential and quadruple-therapy (Fig 8). In this meta-analysis, we found that the eradication rates exhibited by triple-, quadruple- and sequential therapy were 60%, 78%, 86%, respectively, and that probiotic supplementation increased these eradication rates to 82%, 81%, 88%, respectively. Eradication rates differed among different therapy regimens; thus, probiotic-induced effects may not have been observed in cases in which the eradication rate exhibited by antibiotic therapy alone was already high. As quadruple- and sequential therapy exert strong therapeutic effects, it is likely that probiotic supplementation did not significantly increase eradication rates among patients treated with these regimens. However, due to the relatively small numbers of patients treated with quadruple- and sequential therapy in our study, additional and larger trials are needed to confirm our conclusion. We also examined whether probiotic supplementation reduced the incidence of antibiotic-related side effects and found that probiotic supplementation did not decrease the incidence of side effects in patients treated with quadruple-therapy and 14-day therapy (Table 2). We concluded that although probiotic supplementation could not reduce the adverse effects caused by prolonged and high-dose antibiotic treatment in this study, the effect of probiotic supplementation on the incidence of antibiotic-related adverse effects should be examined further in additional studies. Fourteen-day treatment was the best time-frame for achieving high eradication rates but was negatively impacted by serious adverse events and high treatment costs, namely, poor medication tolerance, poor compliance, and drug withdrawal, resulting in reduced eradication rates. In this study, we noted that the 7-day and 14-day therapy eradication rates were 70% and 71%, respectively. After probiotic supplementation, these eradication rates increased to 84% and 80%, respectively. We also found that probiotic supplementation in patients treated with quadruple-therapy and 14-day therapy did not decrease the incidence of side effects, while probiotic supplementation in patients treated with triple-therapy and 7-day therapy significantly increased eradication rates and reduced the incidence of adverse reactions. Thus, we believe that probiotic supplementation in patients treated with 7-day triple-therapy may be the first-choice anti-*H*. *pylori* regimen for patients who cannot afford or cannot tolerate the serious adverse events associated with quadruple- and sequential therapy.

Our analysis showed that using multi-strain probiotics may be more beneficial than using *Lactobacillus* alone with respect to decreasing the overall incidence of eradication regimen-induced side effects (Fig 10, Table 2). The metabolites of lactic acid bacteria have strong antibacterial effects on gram-positive and gram-negative bacteria and can enhance animal humoral and cellular immunity. *Bacillus subtilis* maintains the anaerobic environment of the intestine, inhibits aerobic bacteria growth, and maintains intestinal flora balance. Yeast strains contain a variety of nutrients and improve immunity. *Bifidobacterium* increases the acidity of the intestinal environment, stimulates intestinal peristalsis, and reduces the number of harmful bacteria. Therefore, mixed probiotic use may significantly reduce the incidence of treatment-related adverse reactions. The findings from our study are consistent with those reported by Zhi Fa LV *et al*. [69]. However, the optimal length and timing of supplementation therapy are still uncertain [70]. Our study determined that one week of simultaneous probiotic supplementation and eradication treatment may be ideal both economically and therapeutically (Table 2). However, other factors, such as patient age and region, should also be considered when choosing treatments. It is generally thought that concurrent probiotic and eradication therapy administration is more efficacious than other treatment regimens; however, clinical trials have reported different results regarding the appropriate times for administering probiotics [71,72].

Gastrointestinal flora play roles in biological barrier function, nutrition, immunity, tumor suppression, aging and metabolism. Intestinal flora are relatively stable, and antibiotics can modify their composition, leading to gastrointestinal side effects. However, an RCT showed that adding probiotics to *H*. *pylori* eradication therapy can attenuate antibiotic-induced intestinal bacterial flora changes and inhibit drug-resistant bacteria growth [73]. Another study showed that adding probiotics to *H*. *pylori* eradication therapy attenuated changes in antibiotic intestinal flora [27]. However, additional large RCTs are required to confirm these findings.

All the eligible trials featured clear and adequate randomization methods, and their results remained stable, according to the results of our exclusion sensitivity analysis. Antibiotic-related adverse events were commonly experienced by patients undergoing eradication therapy—although they were mild in most cases—usually leading to treatment discontinuation [74]. In our study, we found that the overall incidence of side effects and the individual incidences of symptoms such as diarrhea, nausea and vomiting, and constipation were significantly decreased in the probiotic supplementation group compared with the control group. However, it should be noted that the included studies comprised patients of different ages who lived in different locations and completed different eradication and probiotic treatment regimens, which made interpreting our results difficult.

In 2013, Lv et al. studied the effects of antibiotics on HP infections. Twenty-one articles satisfied their inclusion criteria, but 11 of those studies were of low quality, exhibiting Jadad scale scores <3. Their study analyzed only triple-therapy and limited numbers of probiotic species (*Lactobacillus*, *Bifidobacterium* and *Saccharomyces*). Their subgroup analysis assessed probiotic supplementation timing, eradication regimens (PPI plus amoxicillin and clarithromycin and PPI plus clarithromycin and tinidazole), probiotic supplementation duration, probiotic species, and patient age. In contrast, our study included related articles published through July 2015. Only one of these articles was considered a high-risk article. We analyzed triple-therapy, quadruple-therapy and sequential therapy, as well as more probiotic species. Our subgroup analysis was more comprehensive, as we analyzed eradication and side effect rates according to region, age (adult or children), location (Europe or Asia), eradication regimen (triple-, quadruple- or sequential therapy), eradication therapy duration (seven or fourteen days), probiotic species (*Lactobacillus* alone or multi-strain probiotics), probiotic supplementation duration (one week, two weeks, or four weeks), and probiotic incorporation time (before, during or after standard treatment). Thus, our results are more comprehensive and detailed than those of the abovementioned study.

This analysis used a rigorous search strategy, strict inclusion criteria and a statistical analysis to systematically and comprehensively determine the effects of probiotics on *H*. *pylori* infection eradication therapy. However, several limitations of this study merit consideration. The allocation concealments of some trials were unclear or inadequate, and others lacked adequate information with which to assess their methodology, which may have resulted in an overestimation of the effects of different treatments. Several trials featured small sample sizes. Thus, although this analysis involved many single studies, its results may not reach statistical significance due to its small sample size. Studies involving larger numbers of subjects are needed. This analysis also included no standardized protocols pertaining to treatment regimens or probiotic species, dosages, or administration durations, and none of the selected trials involved patients from Africa or North America. Although probiotic supplementation can increase anti-*H*. *pylori* eradication rates, the mechanism underlying this phenomenon is not clear. Additional well-designed and large-scale trials are required to obtain further evidence regarding the efficacy of probiotics in *H*. *pylori* eradication.

Current evidence suggests that probiotic supplementation during *Helicobacter pylori* eradication treatment may increase eradication rates and decrease the overall incidence of treatment-related adverse events and the incidences of some specific gastrointestinal symptoms. However, our results should be interpreted with caution due to presence of heterogeneity among the trials included in our analysis. Future studies should focus on clarifying which probiotic strains, dosages, and treatment periods are optimal for patients with *H*. *pylori* infection.

## Supporting Information

S1 File PRISMA 2009 checklist. (DOC)

S2 File PRISMA 2009 flow diagram. (DOC)
