# Supplementary material for: Efficacy of Probiotic Supplementation Therapy for Helicobacter pylori Eradication: A Meta-Analysis of Randomized Controlled Trials
Source: PLoS One. 2016 Oct 10;11(10):e0163743. doi: 10.1371/journal.pone.0163743 (PMC5056761; doi:10.1371/journal.pone.0163743)
Supplement: S1 File — (DOC) [file pone.0163743.s001.doc]

| **Section/topic** | **#** | **Checklist item** | **Reported on page #** |
| --- | --- | --- | --- |
| **TITLE** | | |  |
| Title | 1 | Efficacy of Probiotic of Supplementation Therapy in *Helicobacter pylori* Eradication: a Meta-analysis of Randomized-controlled Trials | 1 |
| **ABSTRACT** | | |  |
| Structured summary | 2 | **Background**: Traditional *Helicobacter pylori* (*H.pylori*) eradication therapies typically yield unsatisfactory rates that are accompanied by antibiotics-related side effects. Recently, some studies reported that supplementation of standard therapies with probiotics could improve efficacy and tolerability in *Helicobacter pylori* eradication therapy.  **Objective**: To assess the effect of probiotics supplementation on improving eradication rates and minimizing therapy-associated adverse events in anti-*Helicobacter pylori* regimens.  **Methods**: We sought eligible randomized controlled trials in electronic databases including PubMed, Medline, Cochrane Central Trials and Chinese Biomedical Database published from there inception until July, 2015. Review Manager 5.3 was used for all statistical analysis.  **Conclusion**: Supplementation with probiotics could be effective in improving *H.pylori* eradication rates and alleviating most therapy-related adverse events. | 1-2 |
| **INTRODUCTION** | | |  |
| Rationale | 3 | Some studies suggested that the supplementation of probiotics as an adjuvant to eradication treatments maybe beneficial in improving *Helicobacter pylori* eradication rates and reducing adverse effects^[16-18]^. However these results still remain controversial ^[19]^. Herein, we conducted a meta-analysis to evaluate the role of probiotics in the eradication of *H.pylori* therapy. | 3 |
| Objectives | 4 | Provide an explicit statement of questions being addressed with reference to participants, interventions, comparisons, outcomes, and study design (PICOS). |  |
| **METHODS** | | |  |
| Protocol and registration | 5 | Indicate if a review protocol exists, if and where it can be accessed (e.g., Web address), and, if available, provide registration information including registration number. |  |
| Eligibility criteria | 6 | Inclusion criteria | 4 |
| Information sources | 7 | PubMed, Medline, Cochrane Central Trials and the Chinese Biomedical Database (from inception until July 2015). | 3 |
| Search | 8 | Literature search strategy | 3-4 |
| Study selection | 9 | Inclusion criteria | 4 |
| Data collection process | 10 | Data extraction | 5 |
| Data items | 11 | List and define all variables for which data were sought (e.g., PICOS, funding sources) and any assumptions and simplifications made. |  |
| Risk of bias in individual studies | 12 | Quality Assessment | 5 |
| Summary measures | 13 | Primary outcome: eradication rates Secondary outcomes: side effects | 6-7 |
| Synthesis of results | 14 | Primary outcome: eradication rates Secondary outcomes: side effects | 6-7 |

Page 1 of 2

| **Section/topic** | **#** | **Checklist item** | **Reported on page #** |
| --- | --- | --- | --- |
| Risk of bias across studies | 15 | Risk assessment of bias of all included trials | 6 |
| Additional analyses | 16 | Subgroup analysis | 7-8 |
| **RESULTS** | | |  |
| Study selection | 17 | Study identification and selection | 4 |
| Study characteristics | 18 | Study identification and selection | 4 |
| Risk of bias within studies | 19 | Risk assessment of bias of all included trials | 6 |
| Results of individual studies | 20 | Primary outcome: eradication rates Secondary outcomes: side effects | 6-7 |
| Synthesis of results | 21 | Primary outcome: eradication rates Secondary outcomes: side effects | 6-7 |
| Risk of bias across studies | 22 | Risk assessment of bias of all included trials | 6 |
| Additional analysis | 23 | Subgroup analysis | 7-8 |
| **DISCUSSION** | | |  |
| Summary of evidence | 24 | Summarize the main findings including the strength of evidence for each main outcome; consider their relevance to key groups (e.g., healthcare providers, users, and policy makers). |  |
| Limitations | 25 | Discuss limitations at study and outcome level (e.g., risk of bias), and at review-level (e.g., incomplete retrieval of identified research, reporting bias). |  |
| Conclusions | 26 | Provide a general interpretation of the results in the context of other evidence, and implications for future research. |  |
| **FUNDING** | | |  |
| Funding | 27 | the fund of luzhou technology Bureau,number: 2015-S-45 |  |

*From:*  Moher D, Liberati A, Tetzlaff J, Altman DG, The PRISMA Group (2009). Preferred Reporting Items for Systematic Reviews and Meta-Analyses: The PRISMA Statement. PLoS Med 6(7): e1000097. doi:10.1371/journal.pmed1000097

For more information, visit: **www.prisma-statement.org**.

Page 2 of 2
