# Supplementary material for: Efficacy of Probiotic Supplementation Therapy for Helicobacter pylori Eradication: A Meta-Analysis of Randomized Controlled Trials
Source: PLoS One. 2016 Oct 10;11(10):e0163743. doi: 10.1371/journal.pone.0163743 (PMC5056761; doi:10.1371/journal.pone.0163743)
Supplement: S2 File — (DOC) [file pone.0163743.s002.doc]

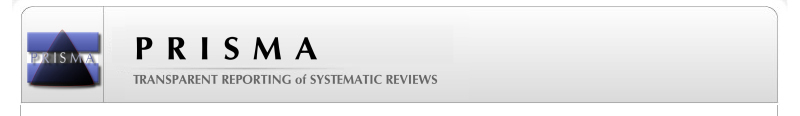
**PRISMA 2009 Flow Diagram**

**Screening**

**Included**

**Eligibility**

**Identification**

Records identified through database searching
(n =1320 )

Additional records identified through other sources
(n = 1051 )

Records after duplicates removed
(n = 759 )

Records screened
(n =473 )

Records excluded
(n =420 )

Full-text articles assessed for eligibility
(n = 53 )

Full-text articles excluded, with reasons
(n =40 )

Studies included in qualitative synthesis
(n = 13 )

Studies included in quantitative synthesis (meta-analysis)
(n = 13 )
